# Genetic Variation of *HvCBF* Genes and Their Association with Salinity Tolerance in Tibetan Annual Wild Barley

**DOI:** 10.1371/journal.pone.0022938

**Published:** 2011-07-28

**Authors:** Dezhi Wu, Long Qiu, Lulu Xu, Lingzhen Ye, Mingxian Chen, Dongfa Sun, Zhonghua Chen, Haitao Zhang, Xiaoli Jin, Fei Dai, Guoping Zhang

**Affiliations:** 1 Agronomy Department, Zhejiang University, Hangzhou, China; 2 College of Plant Science, Huazhong Agricultural University, Wuhan, China; 3 Centre for Plants and Environment, School of Natural Sciences, University of Western Sydney, Richmond, New South Wales, Australia; University of Melbourne, Australia

## Abstract

The evaluation of both the genetic variation and the identification of salinity tolerant accessions of Tibetan annual wild barley (hereafter referred to as Tibetan barley) (*Hordeum vulgare* L. *ssp. Spontaneum* and *H. vulgare* L. ssp. *agriocrithum*) are essential for discovering and exploiting novel alleles involved in salinity tolerance. In this study, we examined tissue dry biomass and the Na^+^ and K^+^ contents of 188 Tibetan barley accessions in response to salt stress. We investigated the genetic variation of transcription factors *HvCBF1*, *HvCBF3 and HvCBF4* within these accessions, conducting association analysis between these three genes and the respective genotypic salt tolerance. Salt stress significantly reduced shoot and root dry weight by 27.6% to 73.1% in the Tibetan barley lines. *HvCBF1*, *HvCBF3* and *HvCBF4* showed diverse sequence variation in amplicon as evident by the identification of single nucleotide polymorphisms (SNPs) and 3, 8 and 13 haplotypes, respectively. Furthermore, the decay of Linkage disequilibrium (LD) of chromosome 5 was 8.9 cM (r^2^<0.1). Marker bpb-4891 and haplotype 13 (Ps 610) of the *HvCBF4* gene were significantly (P<0.05) and highly significantly (P<0.001) associated with salt tolerance. However, *HvCBF1* and *HvCBF3* genes were not associated with salinity tolerance. The accessions from haplotype 13 of the *HvCBF4* gene showed high salinity tolerance, maintaining significantly lower Na^+^/K^+^ ratios and higher dry weight. It is thus proposed that these Tibetan barley accessions could be of value for enhancing salinity tolerance in cultivated barley.

## Introduction

Soil salinity is a major abiotic stress that severely restricts crop productivity; currently over 6% of the world's total land area is adversely affected by salinity. This includes approximately, 20% of cultivated land and nearly half of all irrigated land [1], [2]. Due to constantly deteriorated quality of irrigation water in agricultural practices and other causes, salinity has become a serious issue, posing a great threat to agricultural sustainability [2], [3]. Barley(*Hordeum vulgare* L.)is the fourth most important cereal crop worldwide, and is amongst the most salinity tolerant crop species [1]. Consequently, barley is frequently used as a model crop in the attempts to understand salinity tolerance in plants.

It is reported that wild barley has developed unique mechanisms for surviving harsh environments, mainly through forming new genetic variations and alleles [4], [5]. Tibetan barley from the Qinghai–Tibet Plateau, China, includes a two-rowed type (*H. vulgare* L. *ssp. spontaneum*) and a six-rowed type (*H. vulgare* L. ssp. *agriocrithum*) and is regarded as the progenitor of cultivated barley [6]. Tibetan barley has been found to show wide genetic variations alongside a high tolerance to drought [7] and salinity stress [8]. On the other hand, Feng et al. for example, evaluated the genetic diversity of Tibetan barley using 30 Simple Sequence Repeat (SSR) markers, identifying 229 alleles from 106 accessions with a value of genetic diversity (HT) of 0.572 [9]. In addition, 10 Inter-Simple Sequence Repeat (ISSR) markers and 11 SSR markers were employed to identify genetic diversity in 45 Tibetan barley accessions and values of HT at 0.227 and 0.126, respectively, were obtained [10]. Nonetheless, the relationship between the variation of tolerance and genetic diversity in Tibetan barley is still unknown. Consequently, investigating the association between the genetic variation and physiological performance in wild populations of crop species could be an essential component for identifying genes and alleles underlying salinity tolerance, so could significantly contribute towards attempts to develop more salinity tolerance cultivars.

High salt concentrations in soils inhibit plant growth through both osmotic stress and ionic toxicity. Salinity stress also results in increased levels of oxygen species (ROS) that results in oxidative stress to plant cells [1], [11]. Plants have developed mechanisms for salinity stress adaptation or tolerance, including tissue tolerance to osmotic stress, regulating Na^+^/K^+^ homeostasis, and Na^+^ exclusion [11], [12]. In particular, plant growth and yield [13], Na^+^ and K^+^ concentrations in tissues [14] and K^+^/Na^+^ discrimination in ion transport systems [15] have been widely used as physiological traits for screening for salinity tolerant genotypes.

Using genetic approaches, many genes have been identified and associated with enhanced salinity tolerance in plant species. These genes are generally divided into three groups, according to their function: (1) genes that enhance osmotic protection and ROS scavenging such as the Pyrroline-5-Carboxylase Synthetase (*P5CS*) [16], Osmoregulatory Trehalose Synthesis (*OTS*) [17] and Mannitol-1-Phosphate Dehydrogenase (*M1PD*) [18] genes; (2) genes involved in Na^+^ and K^+^ transport, including the *HKT* family of genes that are involved in K^+^ transport [19], [20] and the *NHX* family of genes (e.g., *NHX1*) or *SOS* genes (e.g., *SOS1*) involved in Na^+^/H^+^ antiport systems [21]–[23]; (3) regulatory genes such as transcription factors (i.e. *CBF/DREB* family) that function in signaling pathways, regulating the expression of downstream genes [3], [24]. The CBF/DREB1 family genes play an important role in the signal transduction pathways involved in low temperature, drought and salinity tolerance in plants [25]–[27]. Expression of *CBF3* and *CBF4* are rapidly induced by drought and salinity stress, while *CBF1* and *CBF3* are induced by low temperature in a number of crops species [28]–[30]. At least 20 *CBF* genes, classified as subgroup *HvCBF1*, *HvCBF3* and *HvCBF4* have been detected in barley [31]. The barley *HvCBF4* gene, for example, encodes a protein closely homologous to CBF/DREB1 in *Arabidopsis*, *Vitis vinifera* and *Vitis riparia* [28], [29]. Importantly, transgenic over-expression of this gene in rice has been demonstrated to enhance tolerance to drought, high-salinity and low-temperature stress [32].

Salinity tolerance is a complex quantitative trait, so quantitative trait loci (QTLs) mapping is commonly used to identify potential genetic loci that could be related to salinity tolerance. In barley, many QTLs that are involved in salinity tolerance have been detected [33], [34]. Recently, association mapping [also known as linkage disequilibrium (LD) mapping] has been adopted as a molecular genetic tool. This method has a higher mapping resolution on phenotypes and traits at the population level, thus it greatly assists in understanding the associations between molecular markers or SNPs and the phenotypes of individuals within the same population [35], [36]. To date, research work based on LD mapping has been conducted on rice [37], maize [38], wheat [39] and barley [40]. However, there are no studies using LD mapping on the abiotic stress tolerance of Tibetan barley. This is primarily due to difficulties in identifying phenotypes and available markers in barley. Fortunately, DArT (diversity arrays technology) has been developed to generate molecular markers in barley and nearly 3500 markers in the barley consensus map have been shown to be ideal for studying genetic diversity in the species [41], [42].

The primary objectives of this work were therefore to (1) identify the population structure of Tibetan barley; (2) evaluate the genetic variation of salinity tolerance in Tibetan barley by using physiological indicators; (3) examine the genetic variation of *HvCBF* genes among Tibetan barley; and (4) determine the association between the genetic variation of markers or SNPs and the phenotypes of salinity tolerance. This will identify the elite alleles controlling salinity tolerance in Tibetan barley.

## Materials and Methods

### Plant materials

A total of 188 Tibetan barley accessions from Huazhong Agricultural University (China) germplasm collection, including two-rowed and six-rowed types [8], [43], were employed for phenotypic and genotypic evaluation. A salt-tolerant barley cultivar CM72 [14], [15], was used as a control.

### Hydroponics

Seeds were surface sterilized with 3% H_2_O_2_ for 20 min and thoroughly rinsed with distilled water, then germinated in sterilized and oven-dried sand in an incubator (22/18°C, day/night) for 10 days. The responses of Tibetan barley to salinity were studied in a glasshouse with natural light, and a temperature of 18±2°C/day and 8±2°C/night. Uniform ten days old barley seedlings were transferred to 35 L plastic containers filled with hydroponic solution of the following composition: 0.4 mM (NH_4_)_2_SO_4_, 0.6 mM MgSO_4_, 0.1 mM K_2_SO_4_, 0.2 mM KNO_3_, 0.2 mM KH_2_PO_4_, 0.4 mM Ca(NO_3_)_2_, 20 µM Fe-Citrate, 5 µM MnCl_2_, 0.4 µM ZnSO_4_, 0.2 µM CuSO_4_, 50 µM H_3_BO_3_ and 0.6 µM molybdic acid. The pH of the culture solution was adjusted into 6.0 using 1 N HCl, as required. All solution was changed weekly. Salinity was supplied to twenty-day old plants, adding it incrementally by 100 mM NaCl per day to reach a final concentration of 300 mM. Control plants were grown under the same conditions, minus the NaCl. The experiment was carried out in 2009 at the Huajiachi campus, Zhejiang University, China.

### Plant biomass

After three weeks of salt treatment, plants were collected and rinsed with tap water for 3 min to remove surface ions and blotted dry with tissue paper. Shoots and roots were separated and dried at 105°C for 3 h, followed by 80°C for 48 h. The dry weight of shoot and root tissues of 12 plants for each genotype in control or treatment were recorded and their relative dry weight was calculated as the ratio of each treated plant to its respective control.

### Na^+^ and K^+^ contents

Dry shoots and roots were ground and a 0.1 g tissue sample was extracted with 10 ml HNO_3_∶H_2_O (1∶1). Na^+^ and K^+^ contents were determined using flame atomic absorption spectrometry (AA6300, Shimadzu, Japan) according to Hack [44].

### PCR amplification and sequencing

Genomic DNA was extracted from pooled leaf tissue of barley seedlings using a modified CTAB method [45]. The reference sequences of *HvCBF1*, *HvCBF3* and *HvCBF4* genes were obtained from the NCBI database according to the accession number: AY785838, AY785846 and AY785851 (http://www.ncbi.nlm.nih.gov). Primers amplifying full-length coding sequences of three candidate genes were designed using Primer5 (http://www.premierbiosoft.com/) according to the reference sequences. Primers for *HvCBF1* were: forward: 5′CCCTGCTTACACTCCAGCA3′: reverse: 5′AGCTAGCCCCAACACTCCTT3′: for *HvCBF3*: forward: 5′CACACTCTCGCTCAAGCTCA3′: reverse: 5′GCAGAATCATCTGGGAAATCA3′: and for *HvCBF4*: forward: 5′TACTCAACCACGCACTCCAG3; reverse: 5′AGCACAATTGAATCGGATGA3′. Primers were synthesized at Shanghai Majorbio Bio-pharm Technology Ltd, China.

The volume of the PCR reaction was 20 µl and was carried out using the Mutiplex PCR kit (Major-bio, Shanghai, China), according to the manufacturer's instructions. The PCR amplification program started at 95°C/5 min, followed by 35 cycles of 95°C/30 s, 60°C/30 s and 72°C/1 min, with a final extension at 72°C for 10 min, and a 4°C holding temperature. PCR products were amplified using *Pfu* DNA polymerase (Promega, USA) and purified with 1% agar gel with a Gel Extraction Kit (Takara, Japan). They were sequenced twice using an ABI3730XL DNA analyzer (Applied Biosystems Inc., USA).

### Polymorphism and haplotype analysis

The sequences obtained were aligned using ClustalX version 2 and the polymorphism sites were detected [46]. The properties of nucleotide and haplotype diversities were evaluated with Dnasp version 5.0 [47].

### Population structure and association analysis

Genetic diversity was examined using 549 randomly selected barley DArT markers over the genome at Diversity Arrays Technology Pty Ltd, Australia. Data from the genetic polymorphism were used to detect the population structure with the STRUCTURE software version 2.3.3, using an admixture model with five independent replicates of 100,000 Markov Chain iterations [48].

Linkage disequilibrium (LD) plot and genotype/phenotype associations were studied by means of the mixed linear model (MLM) in TASSEL software version 2.0 [49]. The values of squared correlation coefficient (r^2^) and the significance of any LD detected between polymorphic sites (*P*) were evaluated with a Fisher's two-tailed test. Association analysis was based on the genetic variations of *HvCBF* genes and relative dry weights of each Tibetan barley accession.

## Results

### Tibetan barley is highly tolerant to salinity

**Figure 1:**
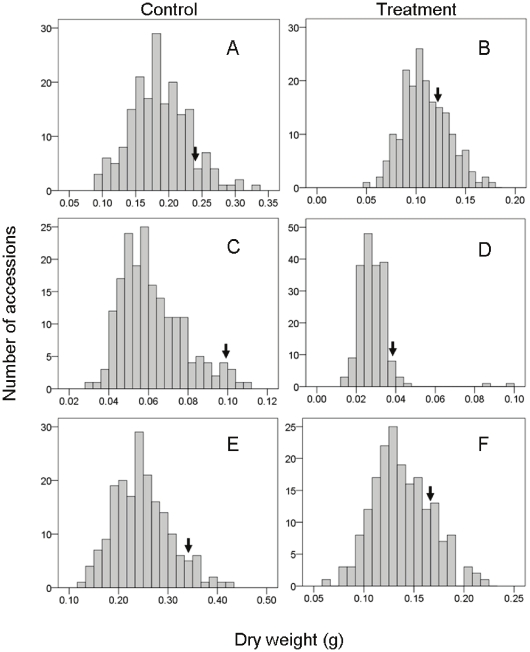
The frequency of dry weight of Tibetan barley. Graphs show the frequency of dry weight (shoot, root or whole-plant) of 188 accessions under control (0 mM NaCl) (A, C and E) or treated (300 mM NaCl) conditions (B, D and F). Arrows indicate the control cultivar, CM72.

**Figure 2:**
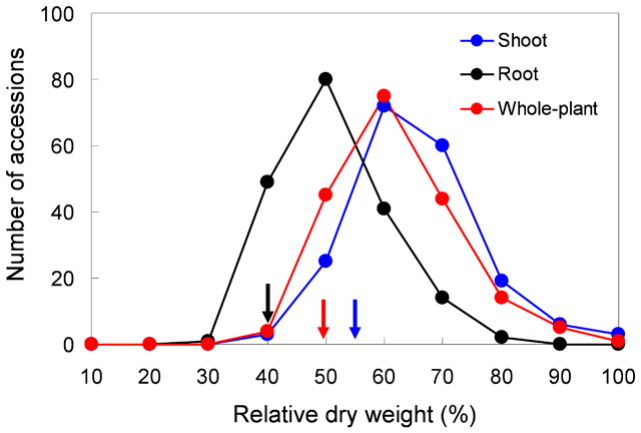
The frequency of relative dry weight of Tibetan barley. The relative dry weight (shoot, root and whole-plant) of 188 accessions was calculated the ratio of the treated (300 mM NaCl) to the control (0 mM NaCl) plants. Arrows indicate the control cultivar, CM72.

The 188 Tibetan barley accessions examined in this study demonstrated a wide range of variation in the shoot, root and whole-plant dry weight in response to 300 mM NaCl treatment (Fig. 1). Taking the combined response of Tibetan barley to salinity stress, Salt stress significantly reduced shoot and root dry weight by 27.6% to 73.1% and the root showed greater reductions in dry weight than the shoot (Fig. 1 and 2). Nonetheless, there were significant differences between the different Tibetan barley lines. Six to thirty-five accessions showed higher shoot, root or whole-plant dry weight than that of CM72 (Fig. 1), a salinity tolerant barley cultivar [14], [15], as a control in this study. Moreover, relative shoot and root dry weight were higher than that of CM72 in 71.3% (134 out of 188 relative shoot weight) and 71.8% (135 out of 188 for root) of Tibetan barley accessions (Fig. 2).

### The Tibetan barley population is consist of eight sub-population

**Figure 3:**
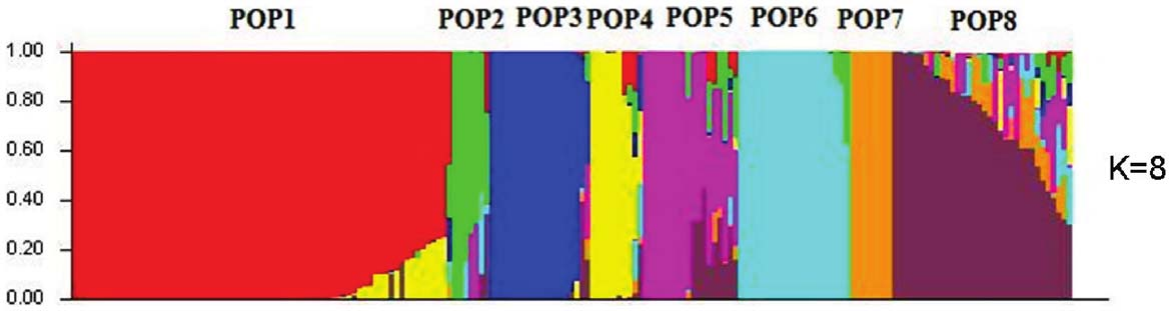
Population structure of Tibetan barley based on genetic diversity detected by 549 DArT markers. Each of the 188 Tibetan barley accessions is denoted as a vertical line, with the eight subgroups represented by different colors.

The effect of population structure should be taken into account and eliminated when association analysis is conducted [50]. In this study, we classified the 188 Tibetan barley accessions into eight sub-populations according to the random distribution of 549 DArT markers over the barley genome, with 72, 7, 19, 9, 18, 21, 8 and 34 accessions for each sub-population (Fig. 3 and Supporting Information S1). The factor of inferred sub-population for each accession, calculated by means of the STRUCTURE software, was used as Q value in later association analysis.

### Nucleotide variation and haplotype diversity

In order to determine any nucleotide variation of *HvCBF1*, *HvCBF3* and *HvCBF4* genes in the Tibean barley population, three polymerase chain reaction (PCR) primers were designed to amplify the whole coding sequence of each of candidate genes. Single nucleotide polymorphisms (SNPs) were detected using sequencing and alignment. We successfully amplified and sequenced *HvCBF1*, *HvCBF3* and *HvCBF4 from* 188, 186 and 188 accessions, respectively. Details of the nucleotide variation and haplotypes of the three genes are presented in Table 1. Two, 15 and 16 SNPs, corresponding to 3, 8 and 13 haplotypes were detected in the three genes, with 0.3, 2 and 2.4 SNPs in each 100 bp of the coding sequence (Table 1). An 18 bp deletion was also identified in *HvCBF3* gene in the amplicon region. Of note, 8 SNPs in *HvCBF3* and 9 SNPs in *HvCBF4* were identified to be non-synonymous polymorphisms (Table 1).

**Table 1:** Single nucleotide polymorphisms (SNPs) and haplotype pattern of *HvCBF* genes in Tibetan barley.

	*HvCBF1* Position (5′ to 3′)			GenBank ID	*HvCBF3* Position (5′ to 3′)																GenBank ID
	162	615	162		28	30	38	51	70	75–92	93	121	123	129	130	304	324	439	451	675	
Reference	G	C	G	AY785838	T	C	C	G	G		C	C	G	G	C	G	C	G	G	C	AY785846
H1	.	.	.	JF796677	.	.	.	.	.		.	.	.	.	.	.	.	.	.	.	JF796669
H2	.	T	.	JF796678	.	.	.	.	.		G	.	.	.	.	A	T	.	C	.	JF796670
H3	A	.	A	JF796679	C	G	T	.	A	del	.	.	.	.	.	.	T	.	C	.	JF796671
H4					C	G	T	.	A	del	.	.	.	.	.	.	T	T	C	.	JF796672
H5					.	.	T	.	.		.	.	.	.	.	.	.	T	.	.	JF796673
H6					.	.	.	.	.		.	.	.	.	.	.	.	T	.	.	JF796674
H7					.	.	.	A	.		G	T	C	A	A	.	.	.	.	A	JF796675
H8					.	.	.	.	.		.	T	C	A	A	.	.	T	.	.	JF796676

	*HvCBF4* Position (5′ to 3′)																GenBank ID
	96	200	201	246	303	351	475	497	534	610	612	616	619	627	629	637	
Reference	G	C	C	C	C	C	G	G	A	C	A	A	C	C	G	G	AY785851
H1	.	.	.	.	.	.	.	.	.	.	.	.	.	.	.	.	JF796660
H2	.	.	.	.	.	.	T	.	.	.	.	.	.	.	.	.	JF796658
H3	.	.	.	.	.	.	T	.	.	.	.	.	.	.	C	.	JF796659
H4	C	.	.	.	T	.	.	C	G	.	.	.	.	.	.	.	JF796664
H5	C	.	.	.	T	.	.	C	.	.	.	.	.	.	.	.	JF796667
H6	.	.	.	.	T	.	.	.	.	.	.	.	.	.	.	.	JF796666
H7	.	.	.	T	.	T	.	.	C	.	.	.	.	.	.	.	JF796657
H8	.	.	.	T	.	T	.	.	.	.	.	.	.	.	.	.	JF796663
H9	.	T	T	.	.	.	.	.	C	.	.	.	.	.	.	.	JF796662
H10	.	.	.	.	.	.	.	.	C	.	.	.	.	.	.	.	JF796665
H11	.	.	.	.	.	.	.	.	C	.	.	.	G	.	C	.	JF796668
H12	.	.	.	T	.	.	.	.	C	.	G	C	G	G	C	C	JF796656
H13	.	.	.	.	.	.	.	.	C	T	G	C	G	G	C	.	JF796661

Note: H: haplotype; del: deletion. Dots indicate the same nucleotide with the reference sequence. The letters in each haplotype represent nucleotide substitution sites and the underlined letters, non-synonymous sites.

We also examined the properties of nucleotide diversity of the three genes in the Tibetan barley population. For the nucleotide diversity of the three genes, *HvCBF3* ranked highest, followed by *HvCBF4* and *HvCBF1*, as indicated by the values of <$>\raster(55%)="rg1"<$> (the possibility of nucleotide being substituted in a population) at 0.0045, 0.0037 and 0.0002. For the haplotype diversity (Hd) of the three genes, *HvCBF4*, *HvCBF3* and *HvCBF1* showed Hd values of 0.777, 0.695, 0.120, respectively.

### Linkage disequilibrium of chromosome 5 and *HvCBF* genes

**Figure 4:**
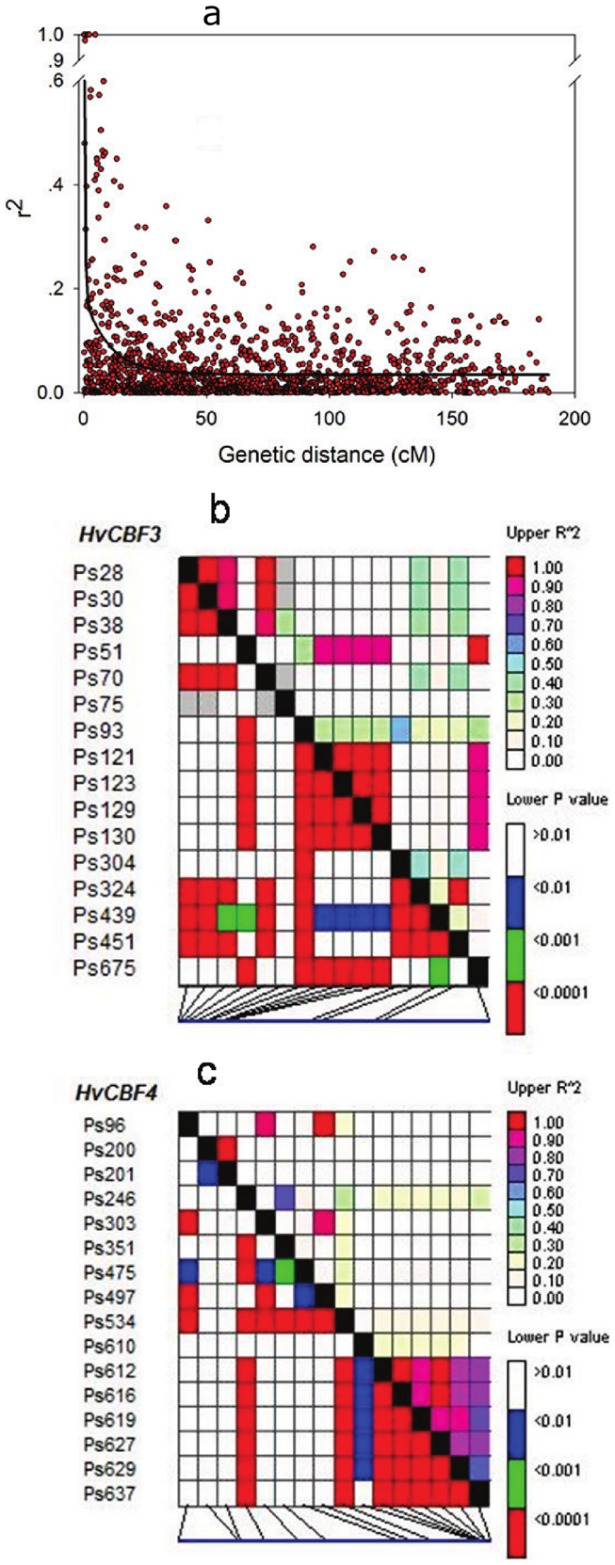
Decay of linkage disequilibrium (LD) of chromosome 5 and intragenic linkage disequilibrium of *HvCBF* genes in Tibetan barley. (a) Decay of linkage disequilibrium of chromosome 5 based on 57 DArT markers, the decay of genetic distance is 8.9 cM (r^2^ = 0.1). The data was fitted to a five-parameter exponential decay model: y = 0.0351+0.9098e^(−2.5845x)^+0.1536e^(−0.0966x)^ (b) intragenic linkage disequilibrium of the *HvCBF3* gene. (c) intragenic linkage disequilibrium of the *HvCBF4* gene; Different colors represent different levels of LD. The labels on the x-axis in Fig. 4b and 4c are in accordance with the SNP on the y-axis in the same order.

Linkage disequilibrium structures of 188 Tibetan barley accessions were investigated using TASSEL software. The linkage disequilibrium structures of chromosome five (Chr.5) were evaluated using 57 DArT markers over this chromosome (Supporting Information S1) and the LD between every two markers was shown in Fig. 4A and Supporting Information S1. Many regions with higher LD values were detected with an r^2^ close to 1 in Chr.5. For instance, the LD decay of genetic distance in Chr.5 was 8.9 cM (r^2^<0.1) or 1.5 cM (r^2^<0.2) (Fig. 4A). On the other hand, the intragenic LD of the *HvCBFs* genes was analyzed between each SNP and the LD structures were found to differ significantly (P<0.01) between SNPs (Fig. 4B, C). Two strong LD blocks from Ps 93 to Ps 130 (37 bp) region in *HvCBF3* and from Ps 612 to Ps 637 (25 bp) region in *HvCBF4*, were found and both demonstrated r^2^ values greater than 0.70 (P<0.01) between SNPs.

### Association analysis between genotype and phenotype

Fifty-seven DArT markers on Chr.5 and non-synonymous SNPs of *HvCBF* genes were used in association analysis between genotypes and relative dry weight. Twenty-three and three DArT markers showed an association with relative dry weight, without or with considering population structure, respectively (Tables 2 and 3). For considering the population structure, marker bpb-4891 was significantly associated with plant weight, explaining the 2.2% and 2.3% phenotypic variation of the relative shoot and whole-plant dry weight respectively. Similarly, marker bpb-2425 was associated with relative root dry weight, explaining 2.0% of the total variation. In contrast, *HvCBF3* and *HvCBF4* genes were closely linked with marker bpb-4891 according to the reported barley genetic map [51].

**Table 2:** The results of DArT markers on chromosome 5 and SNPs of *HvCBF* genes associated with relative dry weight of Tibetan barley.

Traits	Markers/SNPs	F	*p*	R^2^(Model)	R^2^(Marker)
Relative shoot dry weight	bPb-4891	6.66*	0.011	0.037	0.037
	bPb-2960	4.78*	0.030	0.051	0.026
	*HvCBF4*-Ps 610	15.02**	1.48×10^−4^	0.076	0.076
Relative root dry weight	bPb-0085	5.40*	0.021	0.030	0.030
	bPb-0837	13.60**	3.0×10^−4^	0.071	0.071
	bPb-9327	4.74*	0.031	0.027	0.027
	bPb-6710	10.36**	0.002	0.055	0.055
	bPb-5584	6.34*	0.013	0.036	0.036
	bPb-4891	10.22**	0.002	0.055	0.055
	bPb-2425	10.62**	0.001	0.056	0.056
	bPb-8101	8.54**	0.004	0.045	0.045
	bPb-6126	8.16**	0.005	0.044	0.044
	bPb-8771	4.14*	0.043	0.023	0.023
	bPb-2960	13.38**	3.3×10^−4^	0.069	0.069
	bPb-7277	4.57*	0.034	0.025	0.025
	bPb-5238	14.28**	2.2×10^−4^	0.075	0.075
	bPb-0171	8.41**	0.004	0.046	0.046
	bPb-6179	4.05*	0.046	0.160	0.020
	bPb-4595	4.66*	0.032	0.167	0.022
	bPb-1965	3.98*	0.048	0.022	0.022
	bPb-2689	5.80*	0.017	0.031	0.031
	*HvCBF4*-Ps 246	6.16*	0.014	0.033	0.033
	*HvCBF4*-Ps 351	5.08*	0.025	0.027	0.027
Relative plant dry weight	bPb-0837	5.54*	0.020	0.030	0.030
	bPb-4891	8.39**	0.004	0.046	0.046
	bPb-2960	4.88*	0.028	0.026	0.026
	*HvCBF4*-Ps 610	12.03**	6.54×10^−4^	0.062	0.062

The population structure is not considered.

Note: Ps: position site from the start codon.

*(*P*<0.05) indicates the marker or SNP significantly associated with traits.

**(*P*<0.01) indicates the marker or SNP highly significantly associated with traits. R^2^ (Model) is the fraction of the total variation explained by the full model. R^2^ (Marker) is the fraction of the total variation explained by the marker.

**Table 3:** The results of DArT markers on chromosome 5 and SNPs of *HvCBF* genes associated with relative dry weight of Tibetan barley.

Traits	Markers/SNPs	F	*p*	R^2^(Model)	R^2^(Marker)
Relative shoot dry weight	bPb-4891	4.00*	0.047	0.058	0.022
	*HvCBF4*-Ps 610	14.96**	1.54×10^−4^	0.103	0.077
Relative root dry weight	bPb-2425	4.07*	0.045	0.161	0.020
	*HvCBF4-*Ps 610	5.06*	0.026	0.174	0.024
Relative plant dry weight	bPb-4891	4.11*	0.044	0.071	0.023
	*HvCBF4-*Ps 610	12.54**	5.10×10^−4^	0.105	0.064

The population structure is considered.

Note: Ps: position site from the start codon.

*(*P*<0.05) indicates the marker or SNP significantly associated with traits.

**(*P*<0.01) indicates the marker or SNP highly significantly associated with traits. R^2^ (Model) is the fraction of the total variation explained by the full model. R^2^ (Marker) is the fraction of the total variation explained by the marker.

Out of the *HvCBF* genes, only *HvCBF4* was associated with phenotypic variation; the other two genes showed no association with phenotype (Tables 2 and 3). Four SNPs were significantly associated with the phenotype when the population structure was not considered, but numbers of SNPs was reduced to three when the population structure was included. Considering the population structure, SNP (Ps 610) of haplotype 13 from the *HvCBF4* gene exhibited highly significant association with shoot (P<0.0001) and whole-plant (P<0.0001) relative dry weight, explaining 7.7% and 6.4% of the variation in shoot and total dry weight, respectively (Table 3). Based on the distance in the barley genetic map, we found that marker bpb-4891, 7.7 cM from bpb-7852 (Supporting Information S1), was closely linked with the *HvCBF4* gene [51]. The integrated results of our association analysis based on DArT markers and SNPs suggest that SNP (Ps 610) of *HvCBF4* could be related to salinity tolerance.

### Phenotyping of salinity tolerant accessions

**Figure 5:**
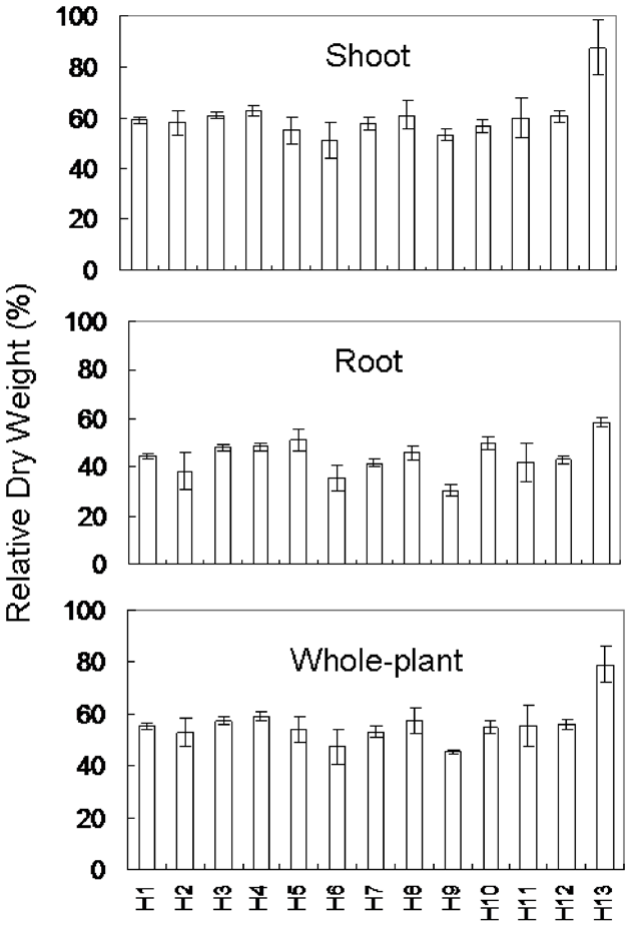
The effects of salt stress on the relative dry weight for thirteen haplotypes. Each bar is the mean of relative dry weight (shoot, root and whole-plant) of accessions belonging to the corresponding haplotype. Error bars are SE.

**Figure 6:**
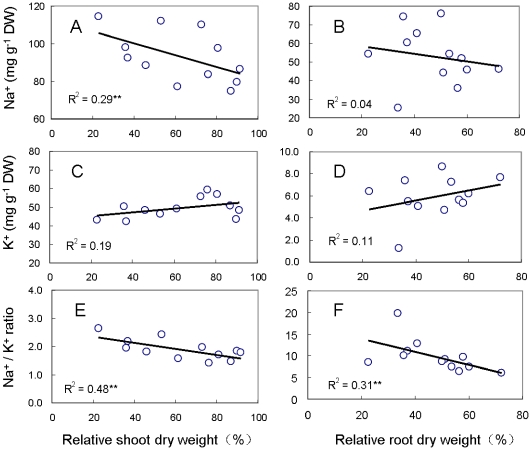
The correlation between relative dry weight and ionic contents. The correlation between Na^+^, K^+^ contents and Na^+^/K^+^ ratios and relative shoot (A, C and E) and root (B, D and F) dry weight is based on four genotypes: CM72, T16, T26 and T169, with three individuals for each genotype.

Relative dry biomass was employed to determine the response of haplotype 13 to salinity stress when compared to the other 12 haplotypes. The relative shoot and whole-plant dry weight differed significantly between all other haplotypes and haplotype 13, which showed on average, a 87.7% and 79.1% higher shoot and whole-plant dry weight. However, a difference in root weight was not detected between haplotype 13 and the rest (Fig. 5). Relative dry weight, tissue Na^+^ and K^+^ contents and the Na^+^/K^+^ ratios were used to evaluate the salinity tolerant Tibetan barley accessions (named T16 and T26) in haplotype 13 of the *HvCBF4* gene, CM72 and salt-sensitive Tibetan barley accession (T169) (Table 4). The Na^+^ content (Fig. 6A) and Na^+^/K^+^ ratios (Fig. 6E) were found to be significantly correlated with relative shoot dry weight, and there was a strong correlation between Na^+^/K^+^ ratio and relative root dry weight (Fig. 6F). In the absence of salinity treatment, Na^+^ and K^+^ contents and the Na^+^/K^+^ ratios in roots did not differ significantly between T16, T26, T169 and CM72, however the K^+^ content in the shoots of Tibetan barley were significantly higher than that of CM72 (Table 4). The reduction of Na^+^ content differed significantly for the four genotypes under salinity stress. T16 and T26 showed 4.2% and 20.8% lower average shoot and root Na^+^ accumulation compared to CM72, and 12.7% and 22.7% lower average shoot and root Na^+^ contents than that in salt-sensitive T169 (Table 4). No difference in shoot K^+^ contents was found between Tibetan barley and CM72, although there was an average 91.1% decrease in response to salinity. Remarkably, Both T16 and T26 exhibited 10.5% and 40.3% lower shoot and root Na^+^/K^+^ ratios than those of CM72 and 26.1% and 28.7% lower average shoot and root Na^+^/K^+^ ratios than those of T169, respectively (Table 4).This strongly indicates that the superior salinity tolerance in Tibetan barley is primarily through Na^+^ exclusion.

**Table 4:** Dry weight (shoot, root and whole-plant), Na^+^ and K^+^ contents and the Na^+^/K^+^ ratios in CM72 and three Tibetan barleys.

	Genotype	DW (g)		Na^+^ (mg g^−1^ DW)		K^+^ (mg g^−1^ DW)		Na^+^/K^+^ ratio	
		shoot	root	shoot	root	shoot	root	shoot	root
CK	CM72	0.24±0.01	0.10±0.01	3.6±0.04	3.2±0.28	43.9±5.69	68.8±19.6	0.08±0.01	0.05±0.01
	T16	0.11±0.01	0.05±0.01	4.3±0.45	3.9±0.21	62.2±2.08	65.3±3.21	0.07±0.01	0.06±0.00
	T26	0.16±0.04	0.05±0.01	3.0±0.19	3.7±0.16	65.6±0.87	69.2±7.71	0.05±0.00	0.05±0.01
	T169	0.19±0.01	0.06±0.01	2.48±0.26	4.3±0.40	62.3±1.27	63.0±9.17	0.04±0.01	0.07±0.01
Salt	CM72	0.13±0.02	0.04±0.01	92.5±10.2	58.5±16.6	48.0±0.92	5.8±2.29	1.9±0.25	12.9±3.52
	T16	0.10±0.01	0.03±0.01	80.2±3.35	45.5±5.35	47.6±2.19	6.9±0.62	1.7±0.11	6.6±0.45
	T26	0.12±0.01	0.03±0.01	97.1±7.65	47.2±2.41	57.4±1.08	5.4±0.41	1.7±0.16	8.8±0.71
	T169	0.06±0.01	0.02±0.01	101.6±6.57	60.0±3.17	45.3±2.55	5.7±0.39	2.3±0.21	10.8±1.27

Data are mean±SE (n = 3). CK: hydroponic solution minus NaCl; Salt: 300 mM NaCl-treated plants.

## Discussion

The Qinghai-Tibet plateau is considered as one of the original centers of cultivated barley [52] and Tibet's harsh environment has influenced its native wild barley populations. This has resulted in a wider genetic variation and much greater stress tolerance compared with cultivated barley [5]. In this study, we used both molecular genetic and physiological techniques to evaluate the variation in the tolerance of 188 Tibetan barley accessions to salinity stress. Salinity tolerance of the Tibetan barley showed a wide genetic variation with many accessions showing a higher salinity tolerance than CM72, a well-known salt-tolerant cultivar. Thus, there must be unique underlying mechanisms, subjected to genetic control, that are involved in the salinity tolerance of Tibetan barley.

In plants, members of *CBFs/DREB* gene family have been found to be critical in the pathways signaling drought, salinity and low temperature stresses [25]–[27]. Furthermore, *CBFs* regulate the expression of many downstream target genes such as *rd29A*, *cor15A* and *kin1* genes that are involved in ROS detoxification [3], [32], [53]. Transgenic over-expression of *AtCBF3* (*DREB1A*) and *HvCBF4* significantly enhances salinity tolerance in salt-susceptible rice [32]. Skinner et al. identified 20 *CBF* genes in barley, genes that belongs to *HvCBF1*, *HvCBF3* and *HvCBF4* subgroup. *HvCBF3* and *HvCBF4* are located in 5H-L of barley genome [31], [51] and are induced by drought and salinity [28], [32].

We selected *HvCBF1*, *HvCBF3* and *HvCBF4* as representative *CBF* genes, revealing their genetic variation in Tibetan barley. Genetic variations in homologous genes in other plants have previously been shown. For instance, the variation of promoter and coding sequence of *CBF1*, *CBF2* and *CBF3* in 48 Arabidopsis accessions had 8.8, 4.5 and 6.2 SNPs per 100 bp in promoter, and 5.1, 3.1 and 4.1 SNPs per 100 bp in coding region, respectively [54], with A higher genetic variation in the promoter regions than in the coding region [54]. Fricano et al. investigated allelic variations of *HvCBF3*, *HvCBF6*, *HvCBF9* and *HvCBF14* in 216 European barley cultivars, landraces and *H. spontaneum* accessions, founding that there were 2.1, 3.1, 1.5 and 2.5 SNPs per 100 bp and 7, 10, 5 and 11 haplotypes respectively, in the coding sequence [55]. Our results showed that the natural genetic variation of *HvCBF3* and *HvCBF4* was as wide in Tibetan barley as that in European barley, even though the *HvCBF1* was mostly invariable. Thus, this study investigated certain details regarding the association between *HvCBF1*, *HvCBF3* and *HvCBF4* genes and salinity tolerance in Tibetan barley.

The level of linkage disequilibrium varies greatly between species and it is also affected by certain factors such as recombination, mutation and selection [56]–[58]. Generally, the LD decay between alleles is slower in inbreds than in outbred lines [59]. In barley, a LD decay of four loci, reaching up to 212 Kb in elite lines when r^2^>0.2, with reduction to 0.4 Kb has been reported in wild lines [40]. Another study showed that the LD decay of 18 loci in wild barley below significant levels within 2 Kb [60]. The average LD within the US barley germplasm decayed over a distance of 20 to 30 cM [61] and in 192 barley accessions from Mediterranean basin the distance of LD decay is reported as 3.62 cM (r^2^<0.2) [62]. In our study, the decay of LD of Chr.5 within the examined Tibetan barley accessions was 8.9 cM (r^2^<0.1) or 1.5 cM (r^2^<0.2). This implies that Tibetan wild barley has a lower LD compared to domesticated barley populations.

In the present study, we analyzed population structure and the associated genetic variation of *HvCBF* genes and a certain complex trait with or without determining the population structure. We found eight sub-populations among the Tibetan barley (Fig. 3 and Supporting Information S1) and a relatively complex population structure existed within the examined Tibetan barley germplasm. Thus, the association results were different between the two methods, with many falsely associated markers or SNPs when the population structure was not considered (Tables 2 and 3). Hence, there is no doubt that the population structure influences the precision of association analysis, and this should be considered when carrying out association studies [63]. Here, we found that marker bpb-4891 and haplotype 13 (Ps 610) of *HvCBF4* gene was significantly associated with salt tolerance. Marker bpb-4891 and bpb-7852 were closely linked with the *HvCBF4* gene, based on the barley physical map at the same location as marker ABC302 according to the reported barley genetic map [51]. Haplotype 13 of *HvCBF4* was associated with relative dry weight (an indicator of salinity tolerance), and SNP (Ps 610) of the *HvCBF4* gene was a non-synonymous mutation that did not change the structure of the gene product. Additionally, no significant association between the genetic variation of *HvCBF1* and *HvCBF3* and relative dry weight was observed (Tables 2 and 3). Thus, determining the combined action of all the transcription factors is importance for comprehensive investigations of associations in barley.

Association mapping has been applied in some crops as a genetic tool with a higher mapping resolution at the population level [37]–[40]. Using LD mapping to detect the association between genetic variation and salinity tolerance in wild populations, such as in the present study is an essential approach for identifying the genes and elite alleles that underlie salinity tolerance and will be of great benefit to barley breeding programs aimed at developing more salinity tolerant cultivars.

Plant biomass, tissue Na^+^ and K^+^ content and the Na^+^/K^+^ ratios have been widely employed as reliable indicators of salinity tolerance in barley [11]–[15]. In terms of dry biomass, haplotype 13 of the *HvCBF4* gene was less affected by salinity stress (Fig. 5). In our case, Tibetan barley accessions named T16 and T26 were identified as highly tolerant to salinity, based on their relative dry weight compared to the other haplotypes (Fig. 5). In addition, Na^+^ and K^+^ contents and the shoot and root Na^+^/K^+^ ratios indicated that these two Tibetan barley accessions were more tolerant to salinity than CM72 (Table 4). According to the known physiological mechanisms of salinity tolerance of CM72 [14], [15], Na^+^ and K^+^ balance and a lower Na^+^/K^+^ ratios in T16 and T26 are attributable to increased salinity tolerance. It is of interest to investigate further the genetic and physiological characteristics of these two Tibetan barley accessions in order to identify the mechanisms underlying their superior salinity tolerance. Such knowledge will increase the potential to develop more salinity tolerant crop species.

## Supporting Information

Supporting Information S1 Supporting figures and tables.
(DOC)
