# Supplementary material for: Genetic Variation of HvCBF Genes and Their Association with Salinity Tolerance in Tibetan Annual Wild Barley
Source: PLoS One. 2011 Jul 28;6(7):e22938. doi: 10.1371/journal.pone.0022938 (PMC3145780; doi:10.1371/journal.pone.0022938)
Supplement: Supporting Information S1 — Supporting figures and tables. (DOC) [file pone.0022938.s001.doc]

# Genetic Variation of *HvCBF* Genes and their Association with Salinity Tolerance in Tibetan Annual Wild Barley

Dezhi Wu^1^, Long Qiu^1^, Lulu Xu^1^，Lingzhen Ye^1^, Mingxian Chen^1^, Dongfa Sun^2^，Zhonghua Chen^3^, Haitao Zhang^1^, Xiaoli Jin^1^, Fei Dai^1^ , Guoping Zhang^1*^

^1^Agronomy Department, Zhejiang University, Hangzhou 310029, China

^2^College of Plant Science, Huazhong Agricultural University, Wuhan 430070, China

^3^Centre for Plants and Environment, School of Natural Sciences, University of Western Sydney, Richmond, 2753 NSW, Australia

^*^Corresponding author; Email: zhanggp@zju.edu.cn


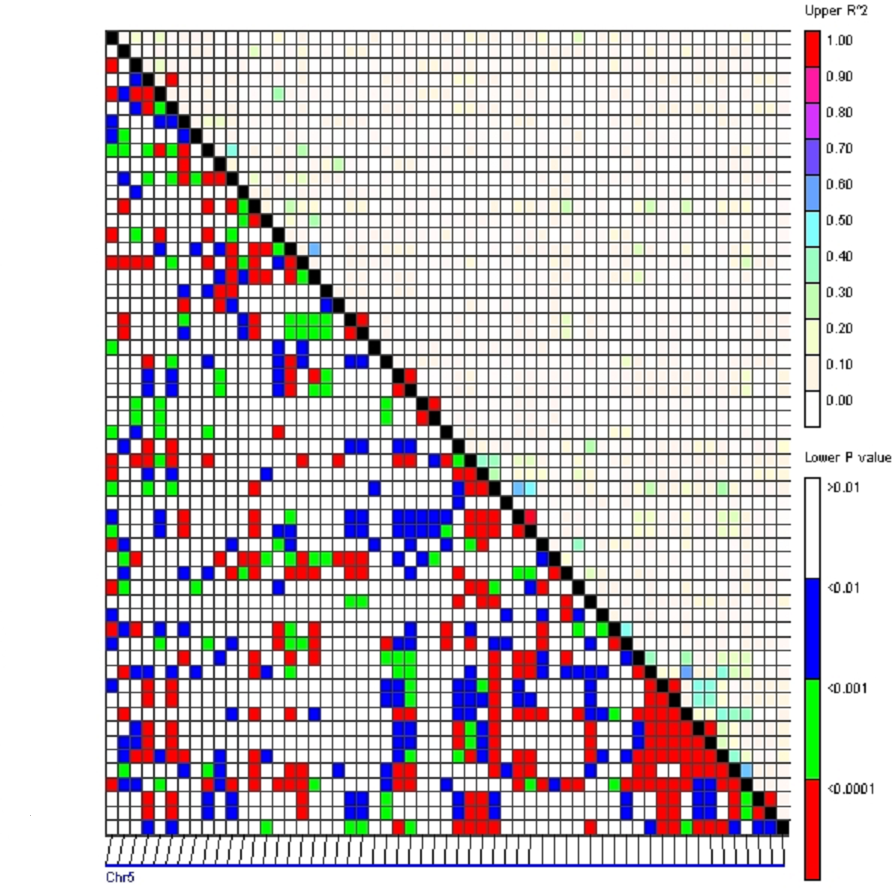


**Chr5**

**Fig.S1.** Linkage disequilibrium of the Chromosome five detected within Tibetan barley by 57 DArT markers. Each point in the LD matrix represents a comparison between a pair of makers. Different colors represent different levels of LD. Above the diagonal are the r^2^ values; below the diagonal are the P values.

**Table S1.** Tibetan barley accessions in each sub-population.

| Sub-Population | Accession number |
| --- | --- |
| Population 1 | T26 T35 T37 T39 T40 T41 T42 T44 T51 T53 T54 T57 T58 T60 T65 T67 T68 T69 T71 T72 T75 T76 T77 T78 T79 T81 T82 T87 T88 T89 T90 T91 T94 T95 T96 T97 T98 T99 T101 T102 T104 T106 T108 T109 T111 T112 T113 T115 T116 T117 T118 T121 T123 T124 T125 T127 T130 T131 T139 T142 T143 T145 T147 T148 T150 T154 T156 T157 T158 T160 T169 T186 |
| Population 2 | T3 T4 T5 T64 T66 T184 T180 |
| Population 3 | T38 T43 T49 T50 T52 T55 T63 T83 T100 T103 T105 T107 T126 T129 T132 T144 T155 T173 T178 |
| Population 4 | T18 T161 T162 T163 T164 T165 T166 T167 T168 |
| Population 5 | T10 T11 T12 T14 T15 T16 T27 T30 T33 T34 T56 T70 T85 T146 T172 T174 T177 T185 |
| Population 6 | T36 T45 T59 T61 T73 T74 T80 T84 T86 T114 T119 T122 T133 T134 T136 T137 T138 T140 T141 T170 T179 |
| Population 7 | T92 T93 T120 T135 T149 T151 T152 T153 |
| Population 8 | T1 T2 T6 T7 T8 T9 T13 T17 T19 T20 T21 T22 T23 T25 T28 T29 T31 T32 T46 T47 T48 T56 T62 T110 T128 T159 T171 T175 T176 T178 T181 T183 T187 T188 |

**Table S2.** Information of fifty-seven DArT markers on Chromosome five.

| Marker | Position | P score | Marker | Position | P score |
| --- | --- | --- | --- | --- | --- |
| bPb-0085 | 1.7 cM | 91.2 | bPb-6126 | 106.9 cM | 78.6 |
| bPb-6051 | 2.6 cM | 83.7 | bPb-0710 | 115.6 cM | 88.8 |
| bPb-8580 | 8.5 cM | 75.3 | bPb-8553 | 120.4 cM | 93.7 |
| bPb-7407 | 16.9 cM | 84.4 | bPb-8771 | 125.7 cM | 88.7 |
| bPb-0837 | 18.0 cM | 88.7 | bPb-4758 | 126.5 cM | 89.8 |
| bPb-6568 | 21.5 cM | 87.3 | bPb-4494 | 127.9 cM | 72.7 |
| bPb-0050 | 31.0 cM | 88.8 | bPb-3700 | 132.9 cM | 82.6 |
| bPb-2900 | 31.8 cM | 88.7 | bPb-8462 | 133.5 cM | 82.2 |
| bPb-9327 | 34.7 cM | 84.9 | bPb-2960 | 134.9 cM | 88.5 |
| bPb-6363 | 36.1 cM | 84.7 | bPb-7404 | 137.8 cM | 81.3 |
| bPb-8589 | 43.5 cM | 88.7 | bPb-1420 | 139.0 cM | 88.1 |
| bPb-2424 | 44.0 cM | 78.5 | bPb-7277 | 139.5 cM | 88.7 |
| bPb-3412 | 45.6 cM | 92.3 | bPb-2006 | 140.7 cM | 74.9 |
| bPb-9306 | 51.0 cM | 76.0 | bPb-3269 | 157.4 cM | 85.7 |
| bPb-6710 | 51.6 cM | 87.5 | bPb-5238 | 159.4 cM | 84.6 |
| bPb-5584 | 54.1 cM | 80.2 | bPb-0171 | 159.9 cM | 88.2 |
| bPb-6260 | 56.8 cM | 92.2 | bPb-6195 | 162.6 cM | 89.2 |
| bPb-4210 | 57.0 cM | 89.5 | bPb-2314 | 163.7 cM | 86.3 |
| bPb-7763 | 71.0 cM | 84.6 | bPb-6179 | 166.1 cM | 90.2 |
| bPb-7852 | 73.6 cM | 80.8 | bPb-4595 | 168.3 cM | 90.5 |
| bPb-7561 | 75.1 cM | 87.8 | bPb-1965 | 171.9 cM | 89.7 |
| bPb-0709 | 76.8 cM | 87.3 | bPb-1719 | 173.7 cM | 85.8 |
| bPb-4891 | 81.3 cM | 77.9 | bPb-0799 | 174.0 cM | 86.6 |
| bPb-6967 | 95.0 cM | 90.3 | bPb-5413 | 177.3 cM | 84.3 |
| bPb-2425 | 97.9 cM | 85.6 | bPb-4971 | 184.3 cM | 91.3 |
| bPb-8101 | 98.2 cM | 84.3 | bPb-2689 | 187.0 cM | 91.6 |
| bPb-1241 | 99.9 cM | 74.8 | bPb-2419 | 188.3 cM | 89.3 |
| bPb-8022 | 101.3 cM | 73.3 | bPb-3973 | 188.8 cM | 91.6 |

Note: cM: centi-Morgen. P score is based on ANOVA measure for markers quality. Marker bPb-7852 is with the same position with marker ABC302 according to Skinner et al. (2006).
